# Research Challenges at the Intersection of Big Data, Security and Privacy

**DOI:** 10.3389/fdata.2019.00001

**Published:** 2019-02-14

**Authors:** Murat Kantarcioglu, Elena Ferrari

**Affiliations:** ^1^Department of Computer Science, University of Texas at Dallas, Richardson, TX, United States; ^2^Department of Theoretical and Applied Science, University of Insubria, Varese, Italy

**Keywords:** big data, security, privacy, cybersecurity, sharing, machine learning

## 1. Overview

As reports from McKinsey Global Institute (Mckinsey et al., 2011) and the World Economic Forum (Schwab, 2016) suggest, capturing, storing and mining “big data” may create significant value in many industries ranging from health care to government services. For example, McKinsey estimates that capturing the value of big data can create $300 billion dollar annual value in the US health care sector and $600 billion dollar annual consumer surplus globally (Mckinsey et al., 2011). Still, several important issues need to be addressed to capture the full potential of big data. As shown by the recent Cambridge Analytica scandal (Cadwalladr and Graham-Harrison, 2018) where millions of users profile information were misused, security and privacy issues become a critical concern. As big data becomes the new oil for the digital economy, realizing the benefits that big data can bring requires considering many different security and privacy issues. This in return implies that the entire big data pipeline needs to be revisited with security and privacy in mind. For example, while the big data is stored and recorded, appropriate privacy-aware access control policies need to be enforced so that the big data is only used for legitimate purposes. On the other hand, while linking and sharing data across organizations, privacy/security issues need to be considered. Below, we provide an overview of novel research challenges that are at the intersection of cybersecurity, privacy and big data.

## 2. Storing and Querying Big Data

One of the ways to securely store big data is using encryption. Once data is encrypted, if the encryption keys are safe, then it is infeasible to retrieve the original data from the encrypted data alone. At the same time, encrypted data must be queried efficiently. Encrypted storage and querying of big data have received significant attention in the literature (e.g., Song et al., 2000; Hacigumus et al., 2002; Golle et al., 2004; Ballard et al., 2005; Chang and Mitzenmacher, 2005; Kantarcıoğlu and Clifton, 2005; Canim and Kantarcioglu, 2007; Shi et al., 2007; Shaon and Kantarcioglu, 2016). Many techniques ranging from simple encrypted keyword searches to fully homomorphic encryption have been developed (e.g., Gentry, 2009). Although there have been major progress in this line of research, breakthroughs are still needed to scale encryption techniques for big data workloads in a cost effect manner. In addition, more practical systems need to be developed for end users. Recent developments that leverage advances in trusted execution environments (TEEs) (e.g., Ohrimenko et al., 2016; Chandra et al., 2017; Shaon et al., 2017; Zheng et al., 2017) offer much more efficient solutions for processing encrypted big data under the assumption that hardware provides some security functionality. Still, the risks of using encrypted data processing (e.g., access pattern disclosure Islam et al., 2012) and TEEs need to be further understood to provide scalability for the big data while minimizing realistic security and privacy risks.

Even if the data is stored in an encrypted format, legitimate users need to access the data. This implies that we need to have effective access control techniques that allow users to access the right data. Although the research community has developed a plethora of access control techniques for almost all of the important big data management systems (e.g., Relational databases Oracle, 2015, NoSql databases Ulusoy et al., 2015a; Colombo and Ferrari, 2018, social network data Carminati et al., 2009) with important capabilities, whether the existing techniques and tools could easily support the new regulatory requirements such as the ones introduced by European Union General Data Protection Directive GDPR (Voigt and Bussche, 2017) is an important question. For example, to address new regulations such as right-to-be-forgotten where users may require the deletion of data that belongs to them, we may need to better understand how the data linked and shared among multiple users in a big data system. For example, multiple users that are tagged in the same picture may have legitimate privacy claims about the picture. This implies that access control systems need to support policies based on the relationships among users and data items (e.g., Pasarella and Lobo, 2017). These observations indicate that understanding how to provide scalable, secure and privacy-aware access control mechanisms for the future big data applications ranging from personalized medicine to Internet of Things systems while satisfying new regulatory requirements would be an important research direction.

## 3. Linking and Sharing Big Data

In many cases, data that belongs to different sources need to be integrated while satisfying many privacy requirements. For example, a patient may visit multiple health care providers and his/her complete health records may not be available in one organization. As another example, passenger data coming from airlines may need to be linked to governmental terrorist watch lists to detect suspicious activity. To protect individual privacy, only the records belonging to government watch lists may be shared. Clearly, these types of use cases require linking potentially sensitive data belonging to the different data controllers. Over the years, private record linkage research has addressed many issues ranging from handling errors (e.g., Kuzu et al., 2013) to efficient approximate schemes that leverage cryptographic solutions (e.g., Inan et al., 2008). Still, the scalability of these techniques for multiple data sources with different privacy and security requirements have not been explored. More research is needed to make these recent developments to be deployed in practice by addressing these scalability issues.

Once data is collected and potentially linked/cleaned, it may be shared across organizations to enable novel applications and unlock potential value. For example, location data collected from mobile devices can be shared with city planners to better optimize transportations networks. Unfortunately, privacy and security issues may prevent such data sharing. Even worse, in some cases such data may be distributed among multiple parties with potentially conflicting interests. For example, different organizations may not want to share their cybersecurity incident data because of the potential concerns where a competitor may use this information for their benefit. Therefore, many issues ranging from security to privacy to incentives for sharing big data need to be considered.

From a privacy point of view, novel privacy-preserving data sharing techniques, based on a theoretically sound privacy definition named differential privacy, have been developed (e.g., Dwork, 2006). These techniques usually work by adding noise to shared data and may not be suitable in some application domains where noise free data need to be shared (e.g., health care domain). In addition, in some cases, these techniques require adding significant amount of noise to protect privacy. This in return may significantly reduce the data utility. On the other hand, some practical risk–aware data sharing tools have been developed (e.g., Prasser et al., 2017). Unfortunately, these practical risk-aware data sharing techniques do not provide the theoretical guarantees offered by differential privacy. Therefore, better understanding of the limits of privacy-preserving data sharing techniques that balance privacy risks vs. data utility need to be developed.

In many cases, misaligned incentives among the data collectors and/or processors may prevent data sharing. For example, instead of getting lab tests conducted by another health care provider, for a hospital, it may be more profitable to redo the tests. To address this type of incentive issues, secure distributed data sharing protocols that incentivize honest sharing of data have been developed (e.g., Buragohain et al., 2003). These protocols usually leverage ideas from economics and game theory to incentivize truthful sharing of big data where security concerns prevent direct auditing (e.g., Kantarcioglu and Nix, 2010; Kantarcioglu and Jiang, 2012). Still addressing incentive issues ranging from compensating individuals for sharing their data (e.g., data market places ^1^) to payment systems for data sharing among industry players need to be addressed. More research that integrates ideas from economics, and psychology with computer science techniques is needed to address the incentive issues in sharing big data without sacrificing security and/or privacy.

## 4. Analyzing Big Data

Another important research direction is to address the privacy and the security issues in analyzing big data. Especially, recent developments in machine learning techniques have created important novel applications in many fields ranging from health care to social networking while creating important privacy challenges.

Again differential privacy ideas have been applied to address privacy issues for the scenarios where all the needed data is controlled by one organization (e.g., McSherry, 2009). These techniques usually require adding noise to the results. Still, it is shown that given large amount of data, these techniques can provide useful machine learning models. To address the scenarios where machine learning models need to be built by combining data that belong to different organization, many different privacy-preserving distributed machine learning protocols have been developed (e.g., Clifton et al., 2003; Kantarcıoğlu and Clifton, 2004; Vaidya and Clifton, 2005). Using cryptographic techniques, these algorithms usually provide security/privacy proofs that show nothing other than the final machine learning models are revealed. Furthermore, these results suggest that most of the privacy-preserving distributed machine learning tasks could be securely implemented by using few basic “secure building blocks” such as secure matrix operations, secure comparison, etc. (Clifton et al., 2003). Still many challenges remain in both settings. In the case of differential private techniques, for complex machine learning tasks such as deep neural networks, the privacy parameters need to adjusted properly to get the desired utility (e.g., classifier accuracy Abadi et al., 2016). The practical implications of setting such privacy parameters need to be explored further. In the case of privacy-preserving distributed machine learning techniques, except few exceptions, these techniques are not efficient enough for big data. Although leveraging trusted execution environments showed some promising results, potential leaks due to side channels need to be considered (Schuster et al., 2015; Costan and Devadas, 2016; Shaon et al., 2017). Therefore, more research is needed to scale these techniques without sacrificing security guarantees.

Unfortunately, securely building machine learning models by itself may not preserve privacy directly. It has been shown that machine learning results may be used to infer sensitive information such as sexual orientation, political affiliation (e.g., Heatherly et al., 2013), intelligence (e.g., Kosinski et al., 2013) etc. Although differential privacy techniques have shown some promise to prevent such attacks, recent results have shown that it may not be effective against many attack while providing acceptable data utility (Fredrikson et al., 2014). These results indicate the need to do more research on understanding privacy impact of machine learning models and whether the models should be built in the first place (e.g., machine learning model that tries to predict intelligence).

## 5. Accountability Issues in Big Data

As machine learning algorithms affect more and more aspects of our lives, it becomes crucial to understand how these algorithms change the way decisions are made in today's data-driven society. The lack of transparency in data-driven decision-making algorithms can easily conceal fallacies and risks codified in the underlying mathematical models, and nurture inequality, bias, and further division between the privileged and the under-privileged (Sweeney, 2013). Although the recent research tries to address these transparency challenges (Baeza-Yates, 2018), more research is needed to ensure fairness, and accountability in usage of machine learning models and big data driven decision algorithms. Understanding the data provenance (e.g., Bertino and Kantarcioglu, 2017) (i.e., how the data is created, who touched it etc.) have shown to improve trust in decisions and the quality of data used for decision making.

In addition to increasing accountability in decision making, more work is needed to make organizations accountable in using privacy sensitive data. With the recent regulations such as GDPR (Voigt and Bussche, 2017), using data only for the purposes consented by the individuals become critical, since personal data can be stored, analyzed and shared as long as the owner of the data consent the data usage purposes. At the same time, it is not clear whether the organizations who collect the privacy sensitive data always process the data according to user consent. An example of this problem is reflected in the recent Cambridge Analytica scandal (Cadwalladr and Graham-Harrison, 2018). In this case, it turns out that the data collected by Facebook is shared for purposes that are not explicitly consented by the individuals which the data belong. As more and more data collected, making organizations accountable for data misuse becomes more critical. It is not clear whether purely technical solutions can solve this problem, even though some research try to formalize purpose based access control and data sharing for big data (e.g., Byun and Li, 2008; Ulusoy et al., 2015b). Legal and economic solutions (e.g., rewarding insiders that report data misuse) need to be combined with technical solutions. Research that addresses this interdisciplinary area emerges as a critical need.

## 6. Blockchains, Big Data Security and Privacy

The recent rise of the blockchain technologies have enabled organizations to leverage a secure distributed public ledger where important information could be stored for various purposes including increasing in transparency of the underlying economic transactions. The first application of Blockchain has been the Bitcoin (Nakamoto, 2008) cryptocurrency. Bitcoin's success has resulted in more than 1000 Blockchain based cryptocurrencies, known as alt-coins.

It turns out that blockchains may have important implications for big data security and privacy. On the one hand, combined with other cryptographic primitives, blockchain based tools (e.g., Androulaki et al., 2018) may enable more secure financial transactions (e.g., Cheng et al., 2018), data sharing (e.g., Kosba et al., 2016) and provenance storage (e.g., Ramachandran and Kantarcioglu, 2018). On the other hand, the data stored on blockchains (e.g., financial transactions stored on Bitcoin blockchain) may be analyzed to provide novel insights about emerging data security issues. For example, it seems that cryptocurrencies are used in payments for human trafficking (Portnoff et al., 2017), ransomware (Huang et al., 2018), personal blackmails (Phetsouvanh and Oggier, 2018), and money laundering (Moser and Breuker, 2013), among many others. Blockchain Data Analytics tools (Akcora et al., 2017) and big data analysis algorithms can be used by law agencies to detect such misuse (for Law Enforcement Cooperation, 2017).

## 7. Adversarial ML and ML for Cybersecurity

Like many application domains, more and more data are collected for cyber security. Examples of these collected data include system logs, network packet traces, account login formation, etc. Since the amount of data collected is ever increasing, it became impossible to analyze all the collected data manually to detect and prevent attacks. Therefore, data analytics are being applied to large volumes of security monitoring data to detect cyber security incidents (see discussion in Kantarcioglu and Xi, 2016). For example, a report from Gartner claims (MacDonald, 2012) that “Information security is becoming a big data analytics problem, where massive amounts of data will be correlated, analyzed and mined for meaningful patterns.” There are many companies that already offer data analytics solutions for this important problem. Of course, data analytics is a means to an end where the ultimate goal is to provide cyber security analysts with prioritized actionable insights derived from big data.

Still, direct application of data analytics techniques to the cyber security domain may be misguided. Unlike most other application domains, cyber security applications often face adversaries who actively modify their strategies to launch new and unexpected attacks. The existence of such adversaries in cyber security creates unique challenges compared to other domains where data analytics tools are applied. First, the attack instances are frequently being modified to avoid detection. Hence a future dataset will no longer share the same properties as the current datasets. For example, attackers may change the spam e-mails written by adding some words that are typically associated with legitimate e-mails. Therefore, the spam e-mail characteristics may be changed significantly by the spammers as often as they want. Secondly, when a previously unknown attack appears, data analytics techniques need to respond to the new attack quickly and cheaply. For example, when a new type of ransomware appears in the wild, we may need to update existing data analytics techniques quickly to detect such attacks. Thirdly, adversaries can be well-funded and make big investments to camouflage the attack instances. For example, a sophisticated group of cyber attackers may create malware that can evade all the existing signature-based malware detection tools using zero day exploits (i.e., software bugs that were previously unknown). Therefore, there is an urgent need to protect machine learning models against potential attacks. Although there is an active research directions for addressing adversarial attacks in machine learning (e.g., Zhou et al., 2012; Szegedy et al., 2013; Goodfellow et al., 2014; Papernot et al., 2016; Zhou and Kantarcioglu, 2016), more research that also leverages human capabilities may be needed to counter such attacks.

## Author Contributions

All authors listed have made a substantial, direct and intellectual contribution to the work, and approved it for publication.

### Conflict of Interest Statement

The authors declare that the research was conducted in the absence of any commercial or financial relationships that could be construed as a potential conflict of interest.

## Footnotes

^1^https://datum.org
